# Constructing an External Control Arm Using the French REALYSA Cohort to Replicate Outcomes of a Randomized Trial Arm in Advanced Hodgkin Lymphoma

**DOI:** 10.1002/cpt.70470

**Published:** 2026-09-07

**Authors:** Olivia Febvey‐Combes, Cédric Rossi, Behrouz Kassai, Tesla Murairi Mirimo, Loïc Chartier, Fanny Cherblanc, Emeline Cugnod, Hervé Ghesquières, Aurélien Belot

**Affiliations:** ^1^ Hospices Civils de Lyon, Pôle de Santé Publique Service Recherche et Epidémiologie Cliniques Lyon France; ^2^ Laboratoire de Biométrie et Biologie Evolutive Université Lyon 1, UMR CNRS 5558 Lyon France; ^3^ Université Bourgogne Europe, CHU Dijon Bourgogne, Hématologie, INSERM1231 CTM Equipe Epi2THM Dijon France; ^4^ Hospices Civils de Lyon, Pôle de Santé Publique Service Hospitalo‐Universitaire de Pharmaco‐Toxicologie Lyon France; ^5^ Centre d'investigation Clinique, INSERM CIC 1407, Groupement Hospitalier Est, Hospices Civils de Lyon Lyon France; ^6^ Lymphoma Academic Research Organisation (LYSARC) Centre Hospitalier Lyon‐Sud Pierre‐Bénite France; ^7^ Département d'hématologie Hospices Civils de Lyon, Centre Hospitalier Lyon‐Sud Pierre‐Bénite France

## Abstract

External control (EC) arms using routine data can be used as comparators in clinical trials under certain conditions. The objective of this study was to assess the ability of an EC arm to replicate outcomes of a randomized controlled trial (RCT) arm receiving the same treatment strategy in advanced stage classical Hodgkin lymphoma and serve as comparator in future trials. Using the prospective real‐world data in lymphoma and survival in adults (REALYSA) cohort, we constructed an EC arm by applying eligibility criteria of the AHL2011 phase III RCT. Propensity score (PS) weighting was used to balance baseline characteristics between the EC arm and the RCT arm that received the same treatment. Progression‐free survival (PFS) and overall response rate (ORR) at the end of first‐line treatment were estimated in both arms. Hazard ratio (HR) for PFS and odds ratio (OR) for ORR were estimated using the EC arm as reference. A sensitivity analysis evaluated PS matching. Four hundred and ten RCT patients and 191 EC patients were analyzed. After PS weighting, patients' characteristics were well‐balanced between arms. PFS estimates were similar in RCT and EC arms with an HR close to 1 (HR, 0.98; 95% CI, 0.59–1.62). ORR estimates were comparable in both arms. Weighting performed better than matching. Using PS weighting gave similar outcomes in the EC arm compared with the RCT arm. Our results suggest that EC studies using routine care data could be considered in the development of strategies for advanced Hodgkin lymphoma.

**Study Highlights**

**WHAT IS THE CURRENT KNOWLEDGE ON THE TOPIC?**

Studies based on external controls (EC) are expanding and may be particularly interesting in rare cancers using methodological precautions. This study assessed for the first time whether an EC arm derived from a cohort of patients treated in clinical practice could be a reliable control group for future trials assessing new strategies in advanced Hodgkin lymphoma.

**WHAT QUESTION DID THIS STUDY ADDRESS?**

This study compared an EC arm derived from the real‐world data in lymphoma and survival in adults (REALYSA) cohort to the experimental arm of the AHL2011 randomized controlled trial (RCT) that received the same treatment strategy. The study focused on two common outcomes in oncology research, that is, progression‐free survival (PFS) and overall response rate (ORR). Different methods were examined to determine whether the EC arm could replicate outcomes of the RCT arm.

**WHAT DOES THIS STUDY ADD TO OUR KNOWLEDGE?**

After applying both trial's eligibility criteria to REALYSA and propensity score (PS)‐based methods to balance RCT and EC arms, similar PFS and ORR outcomes were obtained in the EC arm and in the RCT arm. PS matching was less performant than PS weighting in this context.

**HOW MIGHT THIS CHANGE CLINICAL PHARMACOLOGY OR TRANSLATIONAL SCIENCE?**

This study suggests that EC studies using routine care data and an adapted methodology could be considered in the development of therapeutic strategies for advanced Hodgkin lymphoma.

To assess the efficacy of a new treatment, the randomized controlled trial (RCT) remains the gold standard. In rare diseases involving few patients, conducting an RCT is often a challenge, and single‐arm trials without a randomized control group are frequently performed. This trend is important in oncology when assessing immunotherapies or targeted therapies.^1^, ^2^ However, single‐arm trials have several methodological limitations and are less reliable than RCTs. Moreover, these trials do not estimate the treatment effect because of the absence of an internal control group.^3^

External control (EC) arms may provide useful information in the absence of randomized control arms, enabling a comparison between an experimental arm and a relevant control group.^4^, ^5^, ^6^ These external arms often use routine clinical care data from various sources such as electronic health records, registries, or cohorts. Access to such detailed patient data offers the possibility to construct appropriate comparison arms for patients receiving experimental treatments in single‐arm trials while minimizing costs. These approaches have already been used to support accelerated approvals, especially in rare diseases or in specific sub‐populations, although they raise challenges related to the analysis techniques and the potential relevance of controls (treatment received, time period, etc.).^2^, ^4^, ^7^, ^8^

Before using an EC arm as comparator in clinical trial, it must be determined whether this EC arm is a reliable control arm.^9^ For this purpose, this EC arm can be compared with an RCT arm receiving the same treatment strategy. In the field of oncology, EC arms using routine clinical care data seem in some instances to correctly reproduce the results of RCT arms in non‐small cell lung cancer,^10^, ^11^ but also in other conditions such as metastatic colorectal cancer^12^ or metastatic breast cancer.^13^

Classic Hodgkin lymphoma (cHL) is a rare malignancy, with an incidence of about 1 case per 40,000 people in North America and Europe with a bimodal incidence, which generally develops in young adults (15–35 years old) and in older patients near 70 years old.^14^ For younger patients, HL is a highly curable disease. In this context, it is still crucial to develop new therapeutic strategies that optimize the benefit–risk treatment ratio by maintaining efficacy and reducing toxicity. Moreover, previously prospective trials in advanced cHL required the inclusion of a large number of patients over several years to demonstrate the benefit of one treatment strategy compared with another.^15^, ^16^, ^17^, ^18^, ^19^ The most recent phase III trials such as S1826^19^ and HD21^18^ prospectively included 994 and 1,500 pts, respectively, with long time of recruitment (3 and 4 years, respectively) with an inherent period of follow‐up. Studies using EC arms could allow for early evaluation and identification of promising treatment strategies before moving to phase III.

In the current study, we constructed an EC arm using the cohort named “real‐world data in lymphoma and survival in adults” (REALYSA),^20^ and compared this EC arm to the experimental arm of the AHL2011 trial receiving the same treatment strategy to assess reproducibility.^15^ The AHL2011 trial was a phase III RCT that included 823 cHL patients and evaluated a new strategy guided by the positron emission tomography (PET), which became one of the standards of care especially at the polychemotherapy era to reduce dose intensities without impairing disease control.^15^

## METHODS

### Data sources

#### Clinical trial AHL2011

AHL2011 was a randomized, open‐label, multicenter, non‐inferiority phase III trial in adults with advanced cHL. AHL2011 compared the classical approach (6 cycles of escalated BEACOPP [increased Bleomycin, Etoposide, Doxorubicin, Cyclophosphamide, Vincristine, Procarbazine, Prednisone]) to a de‐escalated approach guided by PET after 2 cycles of eBEACOPP, with patients having a negative PET receiving less intense treatment with 4 cycles of ABVD (Doxorubicin, Bleomycin, Vinblastine, Dacarbazine).^15^ The AHL2011 trial was approved by the relevant authorities and ethics committees in France and Belgium, and all patients provided informed consent. Patients were recruited from May 2011 to April 2014. The study showed that the PET‐guided strategy, with less intense treatment in early responding patients, allowed for similar control of the lymphoma and reduced toxicity.^15^, ^21^ This PET‐guided strategy has become a standard of care for advanced cHL, so it allows comparing this experimental arm to routine recent data of patients treated with the standard of care. In the present work, only data from patients of the experimental arm receiving the PET‐guided strategy were used. We referred to this arm as the RCT arm.

#### REALYSA study

REALYSA was a prospective, multicenter, observational study in patients with lymphoma treated in real clinical practice conditions, conducted across France (NCT03869619).^20^ The REALYSA study was approved by a French ethics committee, and written informed consent was obtained from all patients before data collection. Patients were included between November 2018 and October 2023 with a planned 9‐year follow‐up. REALYSA data export for the present analysis was done in August 2025.

### Study population

Details on the eligibility criteria for AHL2011 have been published.^15^ Briefly, patients aged 16–60 years diagnosed with advanced cHL (Ann Arbor stage III, IV, or IIB at risk with a mediastinum/thorax ratio ≥0.33 or an extra nodal location), who had not been previously treated for their lymphoma, and who underwent a PET scan before the initiation of treatment showing at least one hypermetabolic lesion were included.

### Construction of the external control (EC) arm

A control arm was created using cHL patients who participated in REALYSA to mimic the PET‐guided arm in the AHL2011 trial. First, cHL patients from the REALYSA cohort were selected according to the eligibility criteria of the AHL2011 trial (see **Table** **S1**). Additionally, patients in REALYSA must have received first‐line treatment with eBEACOPP and a PET exam after 2 cycles to be consistent with the PET‐guided strategy of AHL2011.

### Follow‐up period

In the RCT arm, time zero of follow‐up was defined as the date of randomization in the AHL2011 trial.

In the EC arm, time zero was defined as the date of first‐line treatment initiation (i.e., eBEACOPP). Moreover, PET exam after 2 cycles of eBEACOPP (PET2) was used as a proxy for PET‐guided strategy initiation at time zero, which may lead to immortal time bias unless the following assumptions are met: (1) once assigned to the PET‐guided strategy, there are no reasons for a patient not to initiate eBEACOPP other than death, which is unlikely to have occurred in the short time between assignment and start of eBEACOPP; (2) once assigned to the PET‐guided strategy, there are no reasons for a patient not to receive PET2 other than death, disease progression, or toxicity of eBEACOPP. Actually, early deaths and progressions are rare and treatment discontinuations prior to PET2 are exceptional, and none of those were observed in our data used to build the EC arm. Nevertheless, to assess the robustness of these assumptions and a potential immortal time bias (due to selecting patients who had a PET2), a sensitivity analysis was conducted including only patients who underwent the PET2 in both arms, with follow‐up starting at the date of the PET exam.

### Outcomes and assessments

Two major outcomes of the AHL2011 trial were analyzed, that is, progression‐free survival (PFS) (the primary endpoint) and response to treatment (a key secondary endpoint).^15^

PFS was defined as the time from the date of randomization (AHL2011) or the date of initiation of treatment (REALYSA) until the first progression, relapse, or death from any cause. Patients alive and free of progression or relapse were censored at their last follow‐up date.

The overall response rate (ORR) at the end of first‐line treatment was defined as the proportion of patients who achieved a partial response or a complete response. As in the AHL2011 trial, patients without response assessment were considered as nonresponders.

In AHL2011, the following exams were performed: PET scan at baseline, and after two and four cycles of induction chemotherapy; CT after four cycles of induction chemotherapy, at the end of treatment, and every 6 months thereafter until the end of follow‐up.^15^ Moreover, response to treatment was determined as per Cheson 2007 criteria,^22^ based on the investigator's assessment. In REALYSA, response to treatment was based on the hematologist's prescription, in accordance with national and international guidelines for evaluating treatment response by PET‐CT scan.

### Propensity score (PS) weighting

Approaches using PS weighting are based on two steps: (i) estimation of the individual PS, and (ii) estimation of the treatment effect by applying weights defined for our estimand of interest (i.e., the effect of interest in a specific population).

For each patient, PS was calculated patient using multivariable logistic regression. The PS corresponded to the probability of a patient being in the PET‐guided arm from the AHL2011 trial (i.e., RCT arm, thus corresponding to trial participation) given the baseline predetermined covariates. We used 10 covariates and among them those integrated in cHL prognostic score such as International Prognostic Score (IPS)^23^ and more recently in the Advanced‐Stage Hodgkin Lymphoma International Prognostic Index (A‐HIPI).^24^ Baseline covariates included: male sex (Yes/No), age at diagnosis (years), Ann Arbor stage (three levels: I/II, III, IV), Eastern Cooperative Oncology Group Performance Status (ECOG PS) score (three levels: 0, 1, 2), extra nodal sites involved ≥2 (Yes/No), B symptoms (Yes/No), leukocyte count (G/L), lymphocyte count (G/L), hemoglobin level (g/dL), and serum albumin level (g/L).

The PS model was further evaluated by examining key modeling assumptions, including the positivity assumption through the overlap of PS and covariate distributions, the adequacy of the functional form of continuous covariates (i.e., linearity), and the potential presence of clinically relevant interactions between baseline covariates. These diagnostic assessments revealed no evidence of a lack of common support, with 95% of patients falling within the region of PS overlap. In addition, a simple linear specification for continuous covariates was preferred over more complex functional forms (e.g., quadratic terms or splines), and no clinically meaningful interactions between baseline covariates were identified. Collectively, these findings support the adequacy of the final PS model.

The estimand was the average treatment effect on the treated (ATT), where the target population was defined by the population of the AHL2011 clinical trial (i.e., RCT arm). Inverse probability weighting (IPW) was used for estimating the ATT.^25^ In the RCT arm (i.e., patients treated with the PET‐guided strategy in the clinical trial), weights were set to 1 for all patients. In the EC arm (i.e., patients treated with the PET‐guided strategy in clinical practice), weights were computed using the following formula (PS/(1 − PS)) so that the EC arm had the same covariate distribution as the RCT arm.

### Statistical methods

For each outcome, we sought to replicate the estimates obtained from the AHL2011 trial but only for the arm receiving the experimental strategy at that time, which is now a standard of care (i.e., PET‐guided treatment).

First, multiple imputation of missing baseline covariates was implemented using the multiple imputation by chained equations (MICE) method to preserve the number of participants in both RCT and EC arms.^26^ This method assumes that the missing values are missing at random. One imputation model was performed for each outcome, using the R package “mice” with 20 imputations.

Weighting was then performed within each imputed dataset using the function “weightthem” of R package “MatchThem”.^27^ In all datasets, standardized mean differences (SMD) were calculated to assess covariate balance between arms before and after weighting, using the function “bal.tab” of the R package “cobalt.” An absolute SMD <0.1 for a covariate indicated that adequate balance was achieved.^28^ The effective sample size (ESS), which quantifies the loss in precision after weighting, was also examined.^29^

PFS was the first outcome of interest. PFS estimates were obtained using the Kaplan–Meier method, and differences between arms were assessed using the adjusted log‐rank test. Cox proportional hazards model estimated hazard ratio (HR) accounting for IPW‐ATT weights. Estimates in each imputed dataset were pooled using Rubin's rules.^30^ Log‐rank and *P*‐values were pooled according to Li *et al*.^31^ For the Cox model using the EC arm as the reference arm, HR = 1 indicated comparable outcomes in RCT and EC arms, HR < 1 indicated that the hazard of outcome was lower in RCT arm compared with EC arm, and HR > 1 indicated that the hazard of outcome was higher in RCT arm compared with EC arm.

Our primary criterion for establishing reproducibility were balance achievement on baseline covariates and HR not significantly different from 1. Moreover, in an exploratory way, the 95% CI (two‐sided) for the estimated HR was compared with the non‐inferiority margin of the AHL2011 trial (i.e., HR of 1.77).^15^ The upper limit of HR should be lower than 1.77 (and lower limit of HR upper than 0.56 for equivalence) to judge that the difference between our RCT and EC arms was clinically negligible.

Our secondary objective was to replicate ORR of the RCT arm with the EC arm. For comparison of ORR using IPW‐ATT weights, a logistic regression model estimated odds ratio (OR).

In addition to the aforementioned analyses, analyses were also performed before weighting without any adjustment.

### Sensitivity analyses

We performed a sensitivity analysis using PS matching. RCT subjects (corresponding to the “treated”) were matched to EC subjects (corresponding to the “controls”). Since the pool of controls was smaller than treated, common matching without replacement could not match all treated subjects and the estimate may no longer correspond to the ATT.^32^, ^33^ Conversely, matching with replacement permits the same control subject to be matched to multiple treated subjects and that few treated subjects are not matched, and was more suitable in this context.^34^ Two matching algorithms were used: greedy nearest neighbor matching with replacement (ratio 2:1, i.e., 2 controls for 1 treated), (1) without a caliper and (2) with a caliper of width equal to 0.2 of the standard deviation of the logit of the PS as recommended by Austin *et al*.^35^ As for weighting, matching was performed within each imputed dataset using the R package “MatchThem”.^27^ The treatment effects were estimated in each matched dataset accounting for the clustering in the standard error estimation^34^ and pooled according to Rubin's rules.

## RESULTS

### Study population

**Figure 1:**
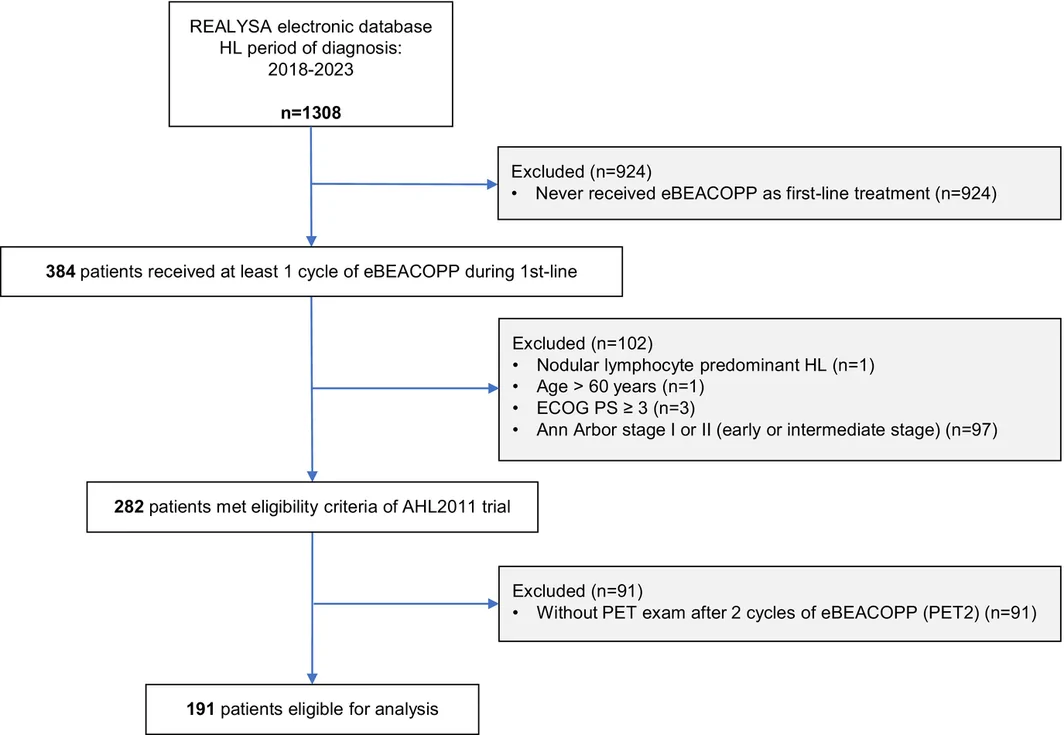
Flow chart for selection of external control (EC) arm of patients with Hodgkin lymphoma (HL). eBEACOPP, escalated Bleomycin, Etoposide, Doxorubicin, Cyclophosphamide, Vincristine, Procarbazine, Prednisone; ECOG PS, Eastern Cooperative Oncology Group Performance Status; HL, Hodgkin Lymphoma; PET, positron emission tomography.

All 410 patients randomized in the PET‐guided arm of the AHL2011 trial were included in the RCT arm. Of 282 patients from REALYSA meeting the eligibility criteria of AHL2011, 191 received a treatment strategy compatible with the PET‐guided arm of AHL2011 and, so, they were included in the EC arm (see Flow chart, **Figure** **1**).

### Baseline characteristics

Baseline covariates of the RCT and EC arm are presented in **Table** **1**. In the RCT arm, 399 of 410 patients (97%) had complete data for the studied baseline characteristics (i.e., no missing data for covariates included in the PS). In the EC arm, 164 of 191 patients (86%) had complete data, and albumin was missing for 12% of EC patients. The majority of participants (61%) were men, the mean age at diagnosis was 32.7 years (SD, 10.7), 532 (89%) had Ann Arbor stage III or IV, and 565 (94%) had ECOG score of 0 or 1 (indicating no or low functional impairment).

**Table 1:** Baseline characteristics

Characteristics	EC arm *N* = 191^a^	RCT arm *N* = 410^a^
Sex		
Female	79 (41%)	157 (38%)
Male	112 (59%)	253 (62%)
Age (years)	33.24 (9.34)	32.46 (11.28)
Ann Arbor stage		
I/II	13 (6.8%)	54 (13%)
III	57 (30%)	114 (28%)
IV	121 (63%)	240 (59%)
Missing	0	2
ECOG PS score		
0	106 (55%)	193 (47%)
1	82 (43%)	184 (45%)
2	3 (1.6%)	31 (7.6%)
Missing	0	2
Extra nodal sites ≥2, Yes	76 (40%)	119 (29%)
B symptoms, Yes	129 (68%)	277 (68%)
Missing	0	2
Leukocytes (G/L)	12.19 (6.31)	12.27 (5.65)
Lymphocytes (G/L)	1.38 (0.85)	1.41 (0.89)
Missing	4	3
Hemoglobin (g/dL)	11.75 (2.05)	11.91 (1.85)
Albumin (g/L)	34.59 (6.33)	35.70 (6.26)
Missing	23	8

EC, External Control; ECOG PS, Eastern Cooperative Oncology Group Performance Status; RCT, Randomized Controlled Trial.

^a^ *n* (%); Mean (SD).

**Figure 2:**
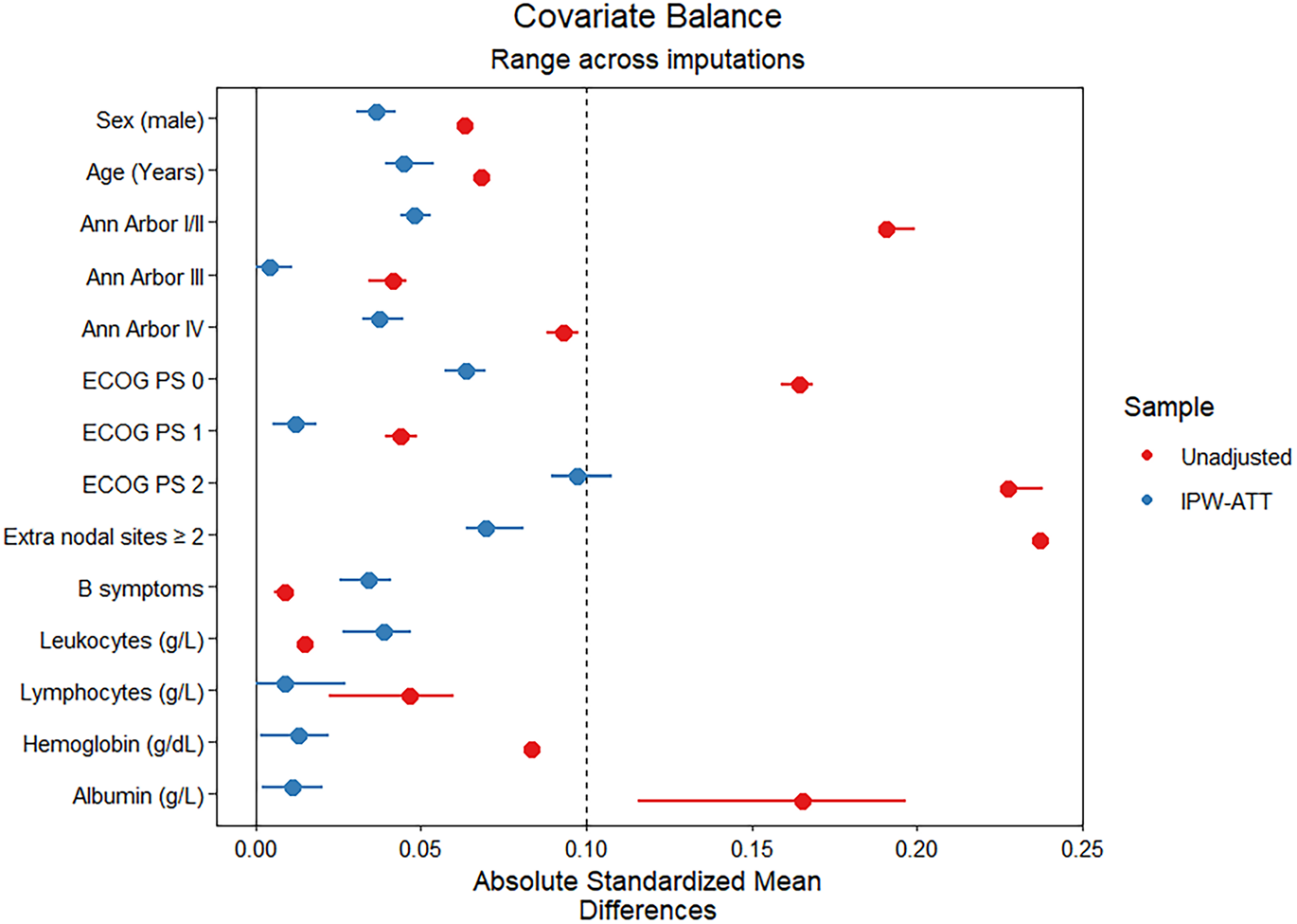
Covariate balance in unadjusted (unweighted) and adjusted (IPW‐ATT) samples. ECOG PS, Eastern Cooperative Oncology Group Performance Status; IPW‐ATT, Inverse Probability Weighting‐Average Treatment effect on the Treated. For each covariate, absolute standardized mean difference (SMD) is averaged (represented by points) and presented with min–max across all imputed datasets (*m* = 20); SMD < 0.1 indicates negligible imbalance between RCT and EC arms.

After multiple imputation to handle missing data and prior to weighting, several covariates were unbalanced between arms (average unadjusted SMD >0.1) (**Table** **2**, **Figure** **2**). In particular, patients from the EC arm were less likely to have an ECOG PS of 2, had more often disease with two or more extra nodal sites involved, and had a lower albumin level.

**Table 2:** Covariate balance before and after adjustment

Covariates	Before adjustment	Weighting (IPW‐ATT)	Matching with replacement	
			Without caliper	With caliper
	RCT patients (*n* = 410) vs. EC patients (*n* = 191)	RCT patients (*n* = 410) vs. weighted EC patients (*n* = 191)	Matched RCT (*n* = 410) vs. matched EC (*n* = 186)	Matched RCT (*n* = 388) vs. matched EC (*n* = 180)
	SMD (min–max)^a^	SMD (min–max)^a^	SMD (min–max)^a^	SMD (min–max)^a^
Sex (male)	0.06 (0.06–0.06)	0.04 (0.03–0.04)	0.07 (0.06–0.09)	0.05 (0.00–0.11)
Age (years)	0.07 (0.07–0.07)	0.04 (0.04–0.05)	0.02 (0.00–0.04)	0.02 (0.00–0.07)
Ann Arbor I/II	0.19 (0.19–0.20)	0.05 (0.04– 0.05)	0.16 (0.16–0.17)	0.02 (0.00–0.05)
Ann Arbor III	0.04 (0.03–0.05)	0.00 (0.00–0.01)	0.07 (0.05–0.10)	0.02 (0.00–0.05)
Ann Arbor IV	0.09 (0.09–0.10)	0.04 (0.03–0.04)	0.04 (0.02–0.07)	0.02 (0.00–0.05)
ECOG PS 0	0.16 (0.16–0.17)	0.06 (0.06–0.07)	0.15 (0.12–0.17)	0.05 (0.00–0.11)
ECOG PS 1	0.04 (0.04–0.05)	0.01 (0.01–0.02)	0.03 (0.00–0.06)	0.07 (0.02–0.13)
ECOG PS 2	0.23 (0.23–0.24)	0.10 (0.09–0.11)	0.22 (0.22–0.23)	0.03 (0.01–0.04)
Extra nodal sites ≥2	0.24 (0.24–0.24)	0.07 (0.06–0.08)	0.11 (0.06–0.13)	0.02 (0.00–0.05)
B symptoms	0.01 (0.01–0.01)	0.03 (0.03–0.04)	0.04 (0.02–0.08)	0.04 (0.00–0.08)
Leukocytes (G/L)	0.01 (0.01–0.01)	0.04 (0.03–0.05)	0.02 (0.00–0.07)	0.05 (0.00–0.10)
Lymphocytes (G/L)	0.05 (0.02–0.06)	0.01 (0.00–0.03)	0.01 (0.00–0.03)	0.02 (0.00–0.08)
Hemoglobin (g/dL)	0.08 (0.08–0.08)	0.01 (0.00–0.02)	0.04 (0.01–0.07)	0.03 (0.00–0.08)
Albumin (g/L)	0.17 (0.12–0.20)	0.01 (0.00–0.02)	0.09 (0.05–0.13)	0.05 (0.01–0.08)

EC, External Control; ECOG PS, Eastern Cooperative Oncology Group Performance Status; IPW‐ATT, Inverse Probability Weighting‐Average Treatment effect on the Treated; RCT, Randomized Controlled Trial; SMD, Standardized Mean Difference.

^a^ Average SMD are presented with min–max across all imputed datasets (*m* = 20); SMD <0.1 indicates negligible imbalance between RCT and EC arms.

After weighting, the ESS of the EC arm was 156 on average across imputations, and characteristics were well‐balanced between the RCT and EC arms (average SMD <0.1 for all covariates) (**Table** **2**, **Figure** **2**).

### PFS outcome

Overall median follow‐up for PFS was 45 months (IQR: 35, 54), with a maximum follow‐up duration of 74 months and 72 months in the RCT and EC arms, respectively. Beyond 60 months of follow‐up, the number of patients that remained at risk of the PFS event dropped in both arms, which limits the interpretation of late survival.

**Figure 3:**
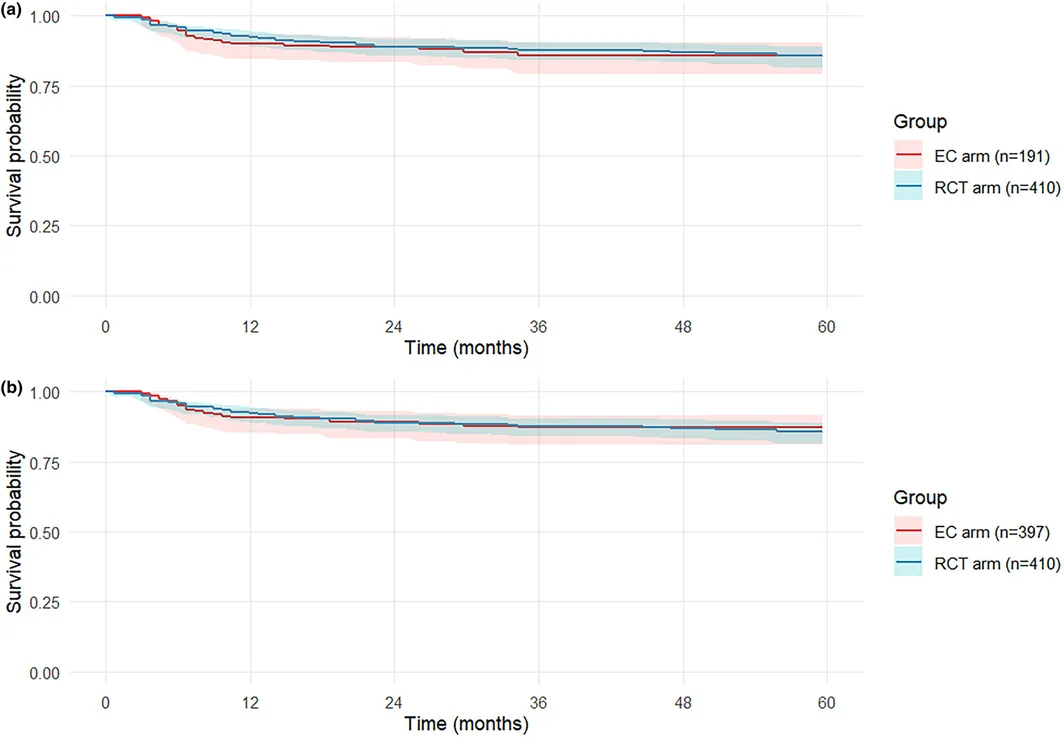
Kaplan–Meier curves comparing progression‐free survival (PFS) for the randomized controlled trial (RCT) arm vs. the external control (EC) arm (pooled data), before weighting **(a)** and after IPW‐ATT weighting **(b)**. IPW‐ATT, Inverse Probability Weighting – Average Treatment effect on the Treated. Sample sizes (pseudo‐population after weighting) are averaged across all imputed datasets (*m* = 20).

PFS events (i.e., progression, relapse or death) occurred in 53 of 410 patients (13%) in the RCT arm and 24 of 191 patients (13%) in the EC arm (median PFS not reached in both arms, **Figure** **3** **a**). The estimated 2‐year PFS was 89.1% in the RCT arm and 88.8% in the EC arm (**Table** **3**). The estimated HR for PFS using EC as reference was 0.89 (95% CI: 0.55–1.47).

**Table 3:** Progression‐free survival (PFS) outcomes before and after weighting (IPW‐ATT)

	Before weighting		After weighting (IPW‐ATT)	
	2‐y PFS (95% CI)	HR (95% CI)^a^	2‐y PFS (95% CI)	HR (95% CI)^a^
RCT arm	89.1 (85.6–91.8)	0.89 (0.55–1.47)	89.1 (85.6–91.8)	0.98 (0.59–1.62)
EC arm	88.8 (83.4–92.6)	–	89.2 (83.4–93.1)	–

Estimates and 95% CI are pooled according to Rubin's rules.

CI, Confidence Interval; EC, External Control; HR, Hazard Ratio; IPW‐ATT, Inverse Probability Weighting‐Average Treatment effect on the Treated; RCT, Randomized Controlled Trial.

^a^ Reference arm is the EC arm.

After weighting, PFS curves of the RCT and the EC arms were not statistically different (adjusted log‐rank *P*‐value = 0.94, **Figure** **3** **b**). The estimated 2‐year PFS was 89.1% in the RCT arm and 89.2% in the EC arm. The estimated HR for PFS was close to 1 (HR, 0.98; 95% CI: 0.59–1.62) (**Table** **3**).

### ORR outcome after the end of first‐line treatment

After a second imputation process including the ORR and application of IPW‐ATT weights, baseline characteristics were well‐balanced between arms (data not shown).

Before weighting, the ORR was similar in the RCT arm (91.0%) and in the EC arm (91.1%) (**Table** **S2**). OR for ORR using EC as reference was 0.98 (95% CI: 0.54–1.80). After weighting, ORR was 91.7% in the EC arm, and OR was 0.91 (95% CI: 0.49–1.70). Again, as expected, the estimated OR was not significantly different from 1.

### Sensitivity analysis

When using matching with replacement (without a caliper restriction), all 410 RCT patients were matched. Of the 191 EC patients, an average of 186 patients (97%) were matched to at least one RCT patient across imputations. Effective sample size was 173 in the EC arm. Some covariates were unbalanced between arms (**Table** **2**, **Figure** **S1**). Therefore, no valid estimation of treatment effect could be performed using this method.

After matching with replacement with a caliper, an average of 388 of 410 RCT patients (95%) and 180 out of 191 EC patients (94%) were matched. Effective sample size was 145 in the EC arm. RCT and EC arms were well‐balanced on average, although there was residual imbalance in some imputations (SMD max slightly above 0.10 for some covariates) (**Table** **2**, **Figure** **S1**). The estimated HR for PFS was 0.86 (95% CI: 0.50–1.50).

## DISCUSSION

Our study explored whether a cohort that included cHL patients treated in routine practice could be useful for the construction of an EC arm. For this purpose, we compared outcomes of an RCT arm of cHL patients receiving the PET‐guided strategy and similar patients receiving the PET‐guided strategy as part of routine clinical care in the REALYSA cohort.

After application of eligibility criteria of the AHL2011 trial, 15% of the HL patients in the REALYSA cohort were eligible for the EC arm. Data on the studied baseline characteristics were missing for 14% of EC patients vs. 3% of RCT patients. Before any adjustment, RCT and EC arms were not balanced for some covariates.

Using PS weighting, balance between RCT and EC arms was improved. Estimates of 2‐year PFS were similar in RCT and EC arms. The HR was very close to 1 (HR, 0.98; 95% CI: 0.59–1.62), indicating that PFS outcomes were comparable in RCT and EC arms. The confidence interval of the HR was quite large due to the low number of events. However, the upper limit of the 95% CI of the HR fell below 1.77 (i.e., the non‐inferiority margin in the AHL2011 trial), suggesting that the difference between the RCT and EC arms could be clinically acceptable. The sensitivity analysis restricted to patients who received PET after cycle 2 yielded effect estimates of similar magnitude and direction to the primary analysis (HR, 0.90; 95% CI: 0.54–1.51). Response rates were also similar in RCT and EC arms with overlapping confidence intervals, but the wide confidence interval of OR prevented formal conclusion on this outcome.

The sensitivity analysis allowed us to compare results obtained using weighting with those obtained using matching, both of which target the ATT estimand. PS matching is a widely used and easy to understand method that aims to balance covariates between groups in non‐randomized studies. Without a caliper, all RCT patients could be matched to EC patients but covariate balance was not achieved and, therefore, no valid estimation was possible. With a caliper restriction, covariate balance was improved although some residual imbalance persisted compared with weighting. In addition, the HR for PFS was lower than with weighting. This result can be partly explained by the reduced sample after the matching procedure that discarded patients in both arms, and the repeated use of some EC patients due to matching with replacement.^34^

A recent literature review reported the results of six studies that directly compared an EC arm to an RCT arm (control or interventional) receiving the same treatment in the field of oncology.^9^ Consistent with our study, EC constructed from different routine care data sources gave similar outcomes to the RCT arm in most cases, although no formal similarity criteria were defined to assess reproducibility. When results were not comparable, the reason was mainly that important data such as eligibility criteria and confounders were not available in the data source used for the EC arm.^36^ In any case, sensitivity analyses testing different methodological approaches appeared essential to strengthen the conclusions of studies based on EC arms, as discussed by Lin *et al*.^9^

PS weighting and matching are two frequently used methods for analyses involving EC arms, which yielded similar results in previous studies with EC.^10^, ^37^ In our study, we observed some discrepancies between the two methods, with weighting performing better than matching, which is consistent with observations from simulation studies.^38^, ^39^ With IPW‐ATT weights, RCT and EC arms were well‐balanced and all patients were part of the analysis. However, effective sample was reduced after weighting, which may have impacted the accuracy of the estimations. In some instances, other estimands may be of interest.^32^ In particular, methods estimating the average treatment effect in the overlap population (ATO), where the target population is an equipoise population (i.e., with very similar characteristics), might have better performances than those targeting other estimands.^40^

The main strength of our study was the use of data from a large randomized phase III study that established a standard of care at time of polychemotherapy era and a prospective national cohort applying a specific data validation process.^41^ In particular, our EC arm was derived from the REALYSA cohort, which demonstrated high‐quality routine data for cHL patients with rare missing data for key variables.^42^ Moreover, in addition to the application of trial's eligibility criteria to construct the EC arm, two adapted PS‐based methods (i.e., weighting and matching) were used to balance baseline characteristics between RCT and EC arms. We also addressed the missing data on the studied covariates using multiple imputation, which is a recommended approach to preserve the number of patients in both arms while accounting for the uncertainty in the missing values.^26^

Our study also has some limitations. First, some eligibility criteria of the AHL2011 trial could not be applied to the REALYSA cohort owing to differences in the examinations in the two contexts, though main prognostic factors were balanced after weighting. Second, we analyzed both survival (PFS) and binary (ORR) outcomes, which are of major interest in the oncology setting. However, methods of measuring these outcomes and the frequency of examinations during follow‐up differed in the two contexts.^41^ Ascertainment of disease progression relied on the clinician's judgment in real clinical practice, whereas the AHL2011 trial used the standardized Cheson criteria,^15^ leading to potential misclassification of outcomes in the EC arm impacting the results.^43^ In addition, unlike the AHL2011 trial, data on interim PET were collected but no formal review of imaging was done in REALYSA, and switching regimens with de‐escalation (eBEACOPP to ABVD) were decided by physicians in each center. Third, the relatively short follow‐up period in our study prevented to reliably estimating long‐term PFS as well as overall survival (OS). However, in a recent study based on nine first‐line trials in cHL, there was a strong correlation between PFS and OS and between treatment effects on PFS and OS, suggesting that PFS outcome is relevant in this indication.^44^ Fourth, the sample size was fixed by design and the study was not powered to answer an equivalence objective. Therefore, the absence of statistically significant differences between RCT and EC arms can be the consequence of an insufficient statistical power, and it does not mean evidence of absence. Moreover, one of our success criteria for establishing reproducibility used the non‐inferiority margin of the AHL2011 trial whose clinical relevance can be disputed in the present work. Finally, due to the absence of randomization and non‐overlap between inclusion periods in the RCT and EC arms, we cannot rule out potential residual confounding that may have affected the results. In particular, we believe that PET interpretation methods have improved between the two periods, but other medical practices have not changed much so this should have a limited impact on our results.

In conclusion, this study explored the possibility of building an EC arm derived from a cohort of patients treated in routine care for advanced Hodgkin lymphoma. Balance and variance are crucial issues in EC studies and must be carefully examined. After applying trial eligibility criteria and PS approach, our findings support the reproducibility of RCT outcomes using the EC arm, that is, our work supports compatibility (rather than validation). Weighting performed better than matching and should be preferred in this context. Trials with EC arms using REALYSA data and an adapted methodology could be considered in the development of new therapeutic strategies in advanced Hodgkin lymphoma.

## FUNDING

This work was supported by the call for projects for young researchers (Year 2024) from the Hospices Civils de Lyon, Lyon, France.

## CONFLICT OF INTEREST

The authors declared no competing interests for this work.

## AUTHOR CONTRIBUTIONS

All authors wrote the manuscript. All authors designed the research. O.F.C., A.B., T.M.M., L.C., and E.C. performed the research and analyzed the data.

## Supporting information


Table S1.
Table S2.
Figure S1.

## Data Availability

Data of the current study are available from the corresponding author on reasonable request.
