# Supplementary material for: Constructing an External Control Arm Using the French REALYSA Cohort to Replicate Outcomes of a Randomized Trial Arm in Advanced Hodgkin Lymphoma
Source: Clin Pharmacol Ther. 2026 Sep 7:10.1002/cpt.70470. Online ahead of print. doi: 10.1002/cpt.70470 (PMC13548820; doi:10.1002/cpt.70470)

Table S1. Applicability of the AHL2011 trial eligibility criteria to REALYSA

AHL2011 ELIGIBILITY CRITERIA	REALYSA
**Inclusion criteria**	**Implementation**
Patient with a first diagnosis of classical Hodgkin lymphoma according to world health organization (WHO) criteria excluding nodular lymphocyte predominant subtype	Yes (according to WHO 2016 classification)
Age of 16 to 60 years	Yes
No previous treatment for Hodgkin lymphoma	Yes
Ann Arbor stages: IIB with mediastinum/thorax ≥0.33 or extra nodal localization, III, IV	Yes
Baseline 18-FDG PET scan (PET0) (F-FDG Positon Emission Tomography) performed before any treatment with at least one hypermetabolic lesion	No
Eastern Cooperative Oncology Group (ECOG) performance status < 3	Yes
With a minimum life expectancy of 3 months	No
Having previously signed a written informed consent	Yes
The patient must be covered by a social security system (in France)	No
**Exclusion criteria**	
Pregnant or lactating women	No
Men and women of childbearing potential not practicing an adequate method of contraception during the study treatment and at least 3 months after the last study drug administration	No
Any history of cancer or cancer treatment during the last 5 years with the exception of non-melanoma skin tumors or stage 0 (in situ) cervical carcinoma	No
Uncontrolled infectious disease, including active HBV (hepatitis B virus) infection defined by either detection of HBs Antigen or presence of anti HBs antibody without detectable anti HBc antibody	No
HIV (Human immunodeficiency virus), HCV (hepatitis C virus) or HTLV (Human T-lymphotropic virus) serology positivity	No
Abnormal liver (bilirubin > 2,5 N) function unless abnormalities are due to Hodgkin lymphoma	No
Abnormal renal (Creatinin > 150 µmol/L) function unless abnormalities are due to Hodgkin lymphoma	No
Leukopenia < 2 G/l or thrombopenia <100 G/l unless abnormalities are due to Hodgkin lymphoma	No
Severe cardio-pulmonary, or metabolic disease interfering with normal application of protocol treatment:<br>o Left Ejection Ventricular Fraction <50%<br>o Respiratory insufficiency prohibiting bleomycin use<br>o Uncontrolled diabetes mellitus leading to impossibility to perform PET scan	No
Impossibility to perform a baseline PET (PET0) before randomization and treatment beginning	No
Incapable person	No

Table S2. Response rate at the end of treatment

	RCT arm<br>N = 410	EC arm<br>N = 191
**Unweighted ORR***	91.0%<br>(95%CI: 87.8%-93.4%)	91.1%<br>(95%CI: 86.1%-94.4%)
**Weighted ORR***	91.0%<br>(95%CI: 87.8%-93.4%)	91.7%<br>(95%CI: 86.8%-94.9%)

*ORR = Complete Response + Partial Response. Patients with “not evaluated” response are included in the denominator in both arms.
Estimates and 95%CI are pooled according to Rubin’s rules.
Abbreviations: CI, Confidence Interval; EC, External Control; ORR, Overall Response Rate; RCT, Randomized Controlled Trial.

Figure S1. Covariate balance in unmatched and matched samples without a caliper (A) et with a caliper (B).

Abbreviations: ECOG PS, Eastern Cooperative Oncology Group Performance Status.
For each covariate, absolute standardized mean difference (SMD) are averaged (represented by points) and presented with min-max across all imputed datasets (m=20); SMD < 0.1 indicates negligible imbalance between RCT and EC arms.

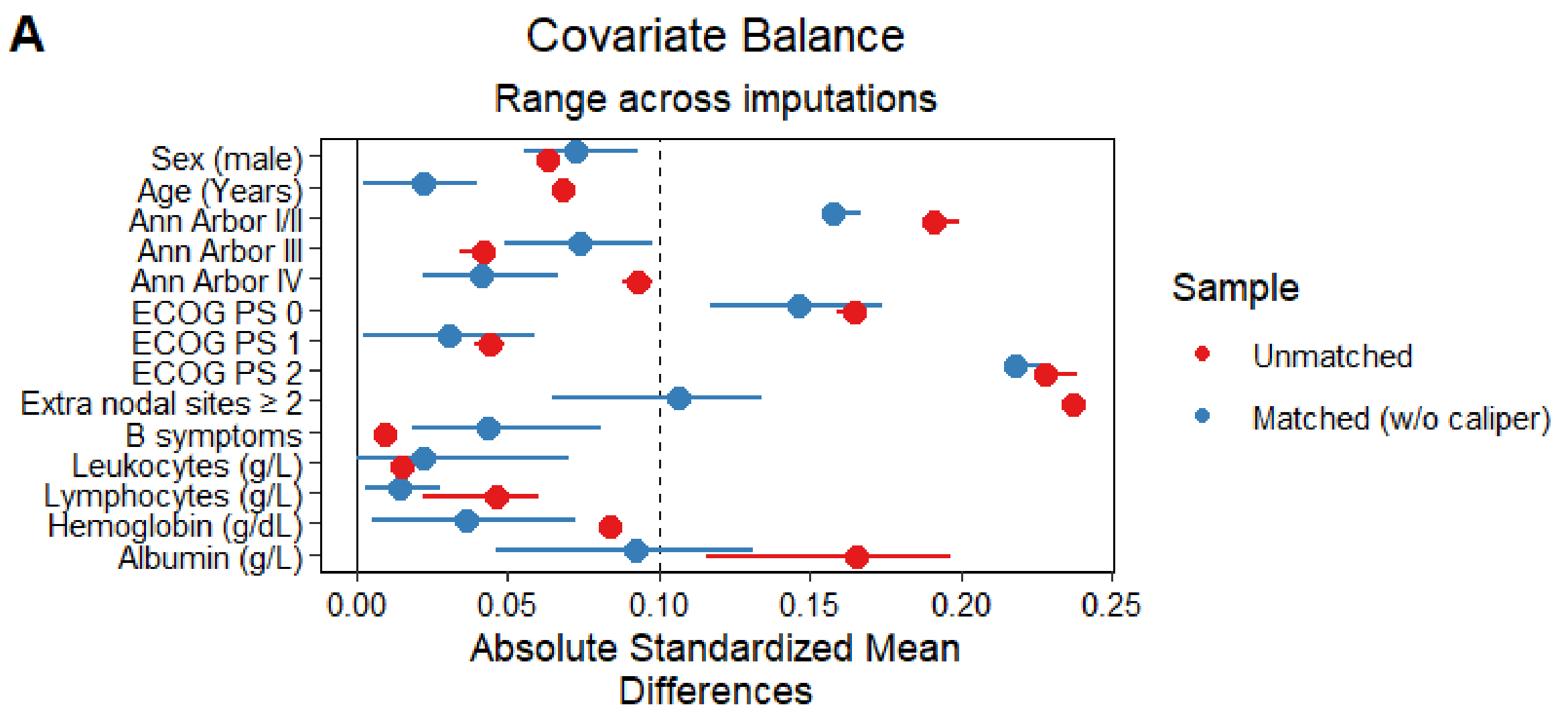

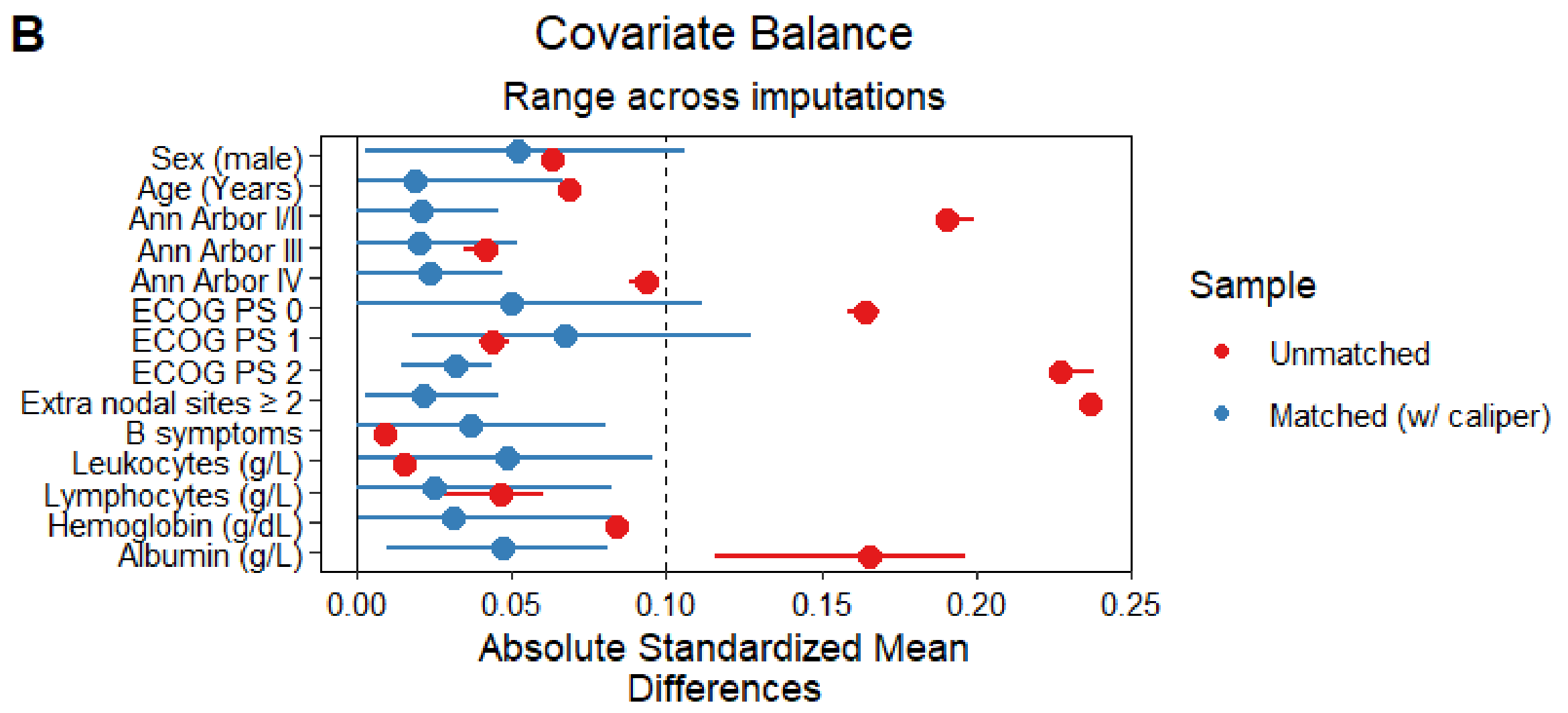

Supplement: Supplementary file 1 [file CPT-9999-0-s001.pdf]
